# Development and characterization of a polarized human endometrial cell epithelia in an air–liquid interface state

**DOI:** 10.1186/s13287-018-0962-6

**Published:** 2018-08-09

**Authors:** Dandan Li, Hui Li, Ying Wang, Ahmed Eldomany, Jing Wu, Chao Yuan, Jing Xue, Juan Shi, Yuanyuan Jia, Chunfang Ha, Shuxia Han, Xiaoming Liu, Jiali Yang, Dan Liu

**Affiliations:** 1 College of Clinical Medicine, Ningxia Medical University, Yinchuan, 750004 Ningxia China; 2 Department of Gynaecology, General Hospital of Ningxia Medical University, Yinchuan, 750004 Ningxia China; 3 Institute of Human Stem Cell Research, General Hospital of Ningxia Medical University, Yinchuan, 750004 Ningxia China; 4 Ningxia Key Laboratory of Clinical and Pathological Microbiology, General Hospital of Ningxia Medical University, Yinchuan, 750004 Ningxia China; 5 College of Life Science, Ningxia University, Yinchuan, 750021 Ningxia China; 6 Key Laboratory of Ministry of Education for Fertility Preservation and Maintenance, Ningxia Medical University, Yinchuan, 750021 Ningxia China

**Keywords:** Endometrium, Epithelial cells, Stem cells, Air–liquid interface, Estrogen

## Abstract

Human endometrial epithelia undergo injury repair and regeneration with the menstrual cycle; however, mechanisms underpinning the roles of endometrial epithelial cells in endometrial lesions and regeneration remain incompletely understood, mainly owing to the difficulty in the isolation and expansion of primary endometrial epithelial cells and the lack of reliable models for *in vitro* and *in vivo* studies. In this report, we sought to improve methods for the isolation and expansion of human endometrial epithelial cells with a Rho-associated protein kinase (ROCK) inhibitor–modified medium and subsequently characterize endometrial epithelium generated with primary cells cultured in an air–liquid interface (ALI) state. Immunocytochemistry staining revealed the expression of epithelial cellular adhesion molecule (EpCam) and stage-specific embryonic antigen-1 (SSEA-1) but a lack of CD13 in endometrial epithelial cells. Meanwhile, a large number of proliferative Ki67^+^ cells were observed in isolated epithelial cells. Importantly, the EpCam^+^/CD13^−^ cells were capable of forming spheroids, a characteristic of epithelial stem/progenitor cells. Interestingly, these cells also exhibited a capacity to reconstitute epithelial layers in an ALI state. Morphological analysis revealed mucosal secretion of differentiated epithelial cells with cilia and microvilli in ALI epithelial cells as determined by electronic microscopy. Immunoblotting assay further demonstrated the expression of endometrial epithelial cell markers keratin 17/19 and EpCam and stem cell marker OCT3/4 but not stromal cell marker Vimentin protein and CD13 in cell expansions. Furthermore, molecular analysis also showed that the exposure of cells to estrogen elevated the expression of estrogen receptor and progesterone receptors in ALI cultures. Our results shed light on the possibility of expanding sufficient numbers of endometrial epithelial cells for stem cell biology studies, and they provide a feasible and alternative model that can recapitulate the characteristics and physiology of endometrial epithelium *in vivo*.

**Electronic supplementary material:**

The online version of this article (10.1186/s13287-018-0962-6) contains supplementary material, which is available to authorized users.

## Introduction

Endometrium is highly regenerative tissue that undergoes a cycle of proliferation, differentiation, shedding, and regeneration 400 times during the menstrual cycle under the control of estrogen or progesterone [1, 2]. It has been demonstrated that endometrial epithelial stem cells play an important role in this repair process and in the integrity and function of endometrium [3, 4]. However, owing to the frequent uterine operation or infection of endometrium, the incidence of endometrial diseases such as intrauterine adhesions has increased in recent years [5] and this has had a severe impact on quality of life for women [6].

Nowadays, our understanding of the biology and function of stem cells in endometrial gland and epithelium is limited by the difficulty in endometrial epithelial cell isolation and culturing, and in stem cell identification and the lack of reliable *in vitro* models. In this report, we described methods for the isolation and culture of human endometrial epithelial cells and characterization of an air–liquid interface (ALI) culture model generated with human endometrial epithelial cells. This study may provide simple and efficient methods for human endometrial epithelial cell isolation and expansion for stem cell biology study, and a reliable and feasible model to recapitulate human endometrium *in vivo*, which can be employed for investigation into the biology and function of human endometrial epithelial stem cells *in vitro*.

## Materials and methods

### Ethnic statement and human endometrial tissue procession

The study and protocol were approved by the ethics committee for conducting human research at the General Hospital of Ningxia Medical University (NXMU-2017-063). All patients analyzed were above 25 years old and were given informed consents. Biopsies of human endometrium samples were obtained from the premenopausal women undergoing hysteroscopy at the General Hospital of Ningxia Medical University. Tissues from 12 donors were analyzed in this study. The endometrium was scraped off and collected into D-Hanks phosphate-buffered saline (PBS) at 4 °C and was subsequently treated for isolation of cells within 2–4 h. Detailed information on materials and methods is provided as supplemental data (Additional file 1), and the antibodies used in this work are listed in Additional file 2.

### Isolation and culture of human endometrial glandular epithelial cells

The isolation of human endometrial epithelial cells was conducted as described in a previous study with minimal modification [7]. Briefly, the human endometrial biopsy was minced with scissors into small pieces of less than 1 mm^3^ before it was washed with PBS containing antibiotics. Subsequently, the minced biopsies were dissociated in a dissociation buffer containing 3.0 mg/mL collagenase type 4 (Sigma-Aldrich, St. Louis, MO, USA) in PBS for 7 min at 37 °C with gentle agitation. Then the same volume of Accumax (Innovative Cell Technologies, San Diego, CA, USA) was added in the dissociated solution and incubated for an additional 7 min at 37 °C with continuous agitation. The digestive reaction was terminated by adding fetal bovine serum to the dissociated fragment/cell suspension in a final concentration of 10%. The dissociated fragment/cell suspension was filtered through a 400-mesh nylon sieve, and residual cell clumps on the sieve were glandular epithelial cells and were washed off with D-Hanks into a tube. The cells were collected by centrifugation (100*g* for 5 min), resuspended in 2 mL of culture medium containing 10 μM of Rho-associated protein kinase (ROCK) inhibitor Y-27632 (Sigma-Aldrich), and seeded onto a 10-cm culture dish pre-coated with 70 μg/mL collagen type I rat tail (BD Biosciences, Franklin Lakes, NJ, USA). The cells were maintained in the culture medium at 37 °C in a humidified environment with 5% CO_2_. The adherent cells were dissociated by using Accutase solution (Millipore, Burlington, MA, USA) at 2–3 days after seeding, and the cells were reseeded at a ratio of 1:3–5 for passage. Other materials and methods are provided as supplemental data in Additional files 1 and 2.

## Results

### Isolation and ALI culture of human endometrial epithelial cells

**Fig. 1:**
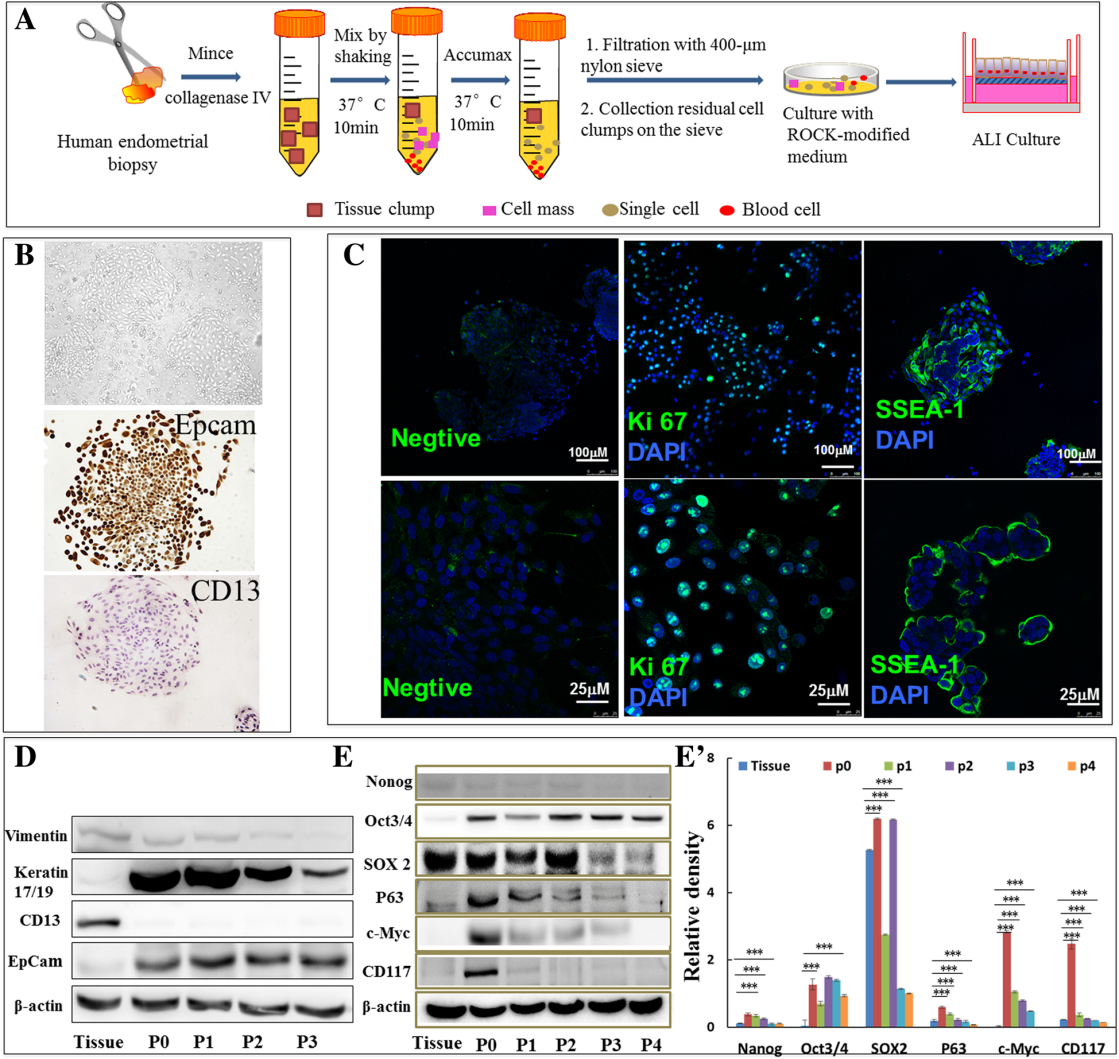
Isolation and identification of endometrial epithelial cells. (**A**) The schematic showed the procedure of isolation of endometrial epithelial cells and generation of an air–liquid interface (ALI) culture. (**B**) Identification of endometrial epithelial cells. Cells grown with Rho-associated protein kinase (ROCK)-modified medium on collagen type I rat tail–coated dishes exhibited a capacity to form colonies, which expressed cell surface antigen epithelial cellular adhesion molecule (EpCam) but not CD13 as determined by an immunocytochemistry assay with hematoxylin counterstaining. (**C**) Immunofluorescent staining for Ki67 or stage-specific embryonic antigen-1 (SSEA-1) (green) revealed that a subset of primary human endometrial epithelial cells expressed Ki67 or SSEA-1. (**D**) Immunoblotting assay confirmed the expression of indicated proteins of interest in native human endometrial biopsy tissues and isolated cell cultures of passage 0–3. (**E**) Immunoblotting assay confirmed stem cell marker expression of Nanog, Oct3/4, Sox 2, p63, c-Myc, and CD117 (c-kit) in native human endometrial biopsy tissues and isolated cell cultures of passages 0–4. (**E′**) Semi-quantitative analysis of the fold changes of the expression of proteins in (**E**) accessed by a densitometric assay. Compared with passage 0 (P0) cells, *P <0.05; **P <0.01 (analysis of variance). Scale bars = 100 μm (**C**) and 25 μm (**C′**)

In this study, we initially isolated epithelial cells from biopsies of human endometrial tissue. The workflow of isolation and culture of epithelial cells is summarized in Fig. 1A. The colonies with morphology of epithelial cells were observed when the initially isolated cells were grown on collagen type I rat tail–coated dishes in ROCK inhibitor–modified medium at 48 h (Fig. 1B). The initially isolated cells (passage 0, P0) were stained with epithelial cell marker epithelial cellular adhesion molecule (EpCam), stroma cell marker CD13, and stem cell marker stage-specific embryonic antigen-1 (SSEA-1). The immunocytochemistry assay revealed that the epithelial cells expressed EpCam but not CD13 (Fig. 1B). Immunocytofluorescent staining further demonstrated the expression of SSEA-1 (Fig. 1C) and a large number of proliferation marker Ki67-positive cells in isolated epithelial cells (Fig. 1C). In addition, immunoblotting assay revealed the expression of epithelial cell markers Keratin 17/19 and EpCam (Fig. 1D) and stem cell markers octamer-binding transcription factor 3/4 (OCT3/4), Sry-box-2 (SOX2), P63, c-Myc, and CD117 (c-kit) during the cell expansion culture (Fig. 1E). Of interest, the expression of SOX2, P63, c-Myc, and CD117 was reduced with the passages of cell cultures (Fig. 1E). Equally noteworthy, although the primary cells could rapidly proliferate to passage 3 (P3), they were senescent in P4 or slowly grown in P4 in current culture conditions (data not shown).

### Morphological analysis of endometrial epithelial cell ALI cultures

**Fig. 2:**
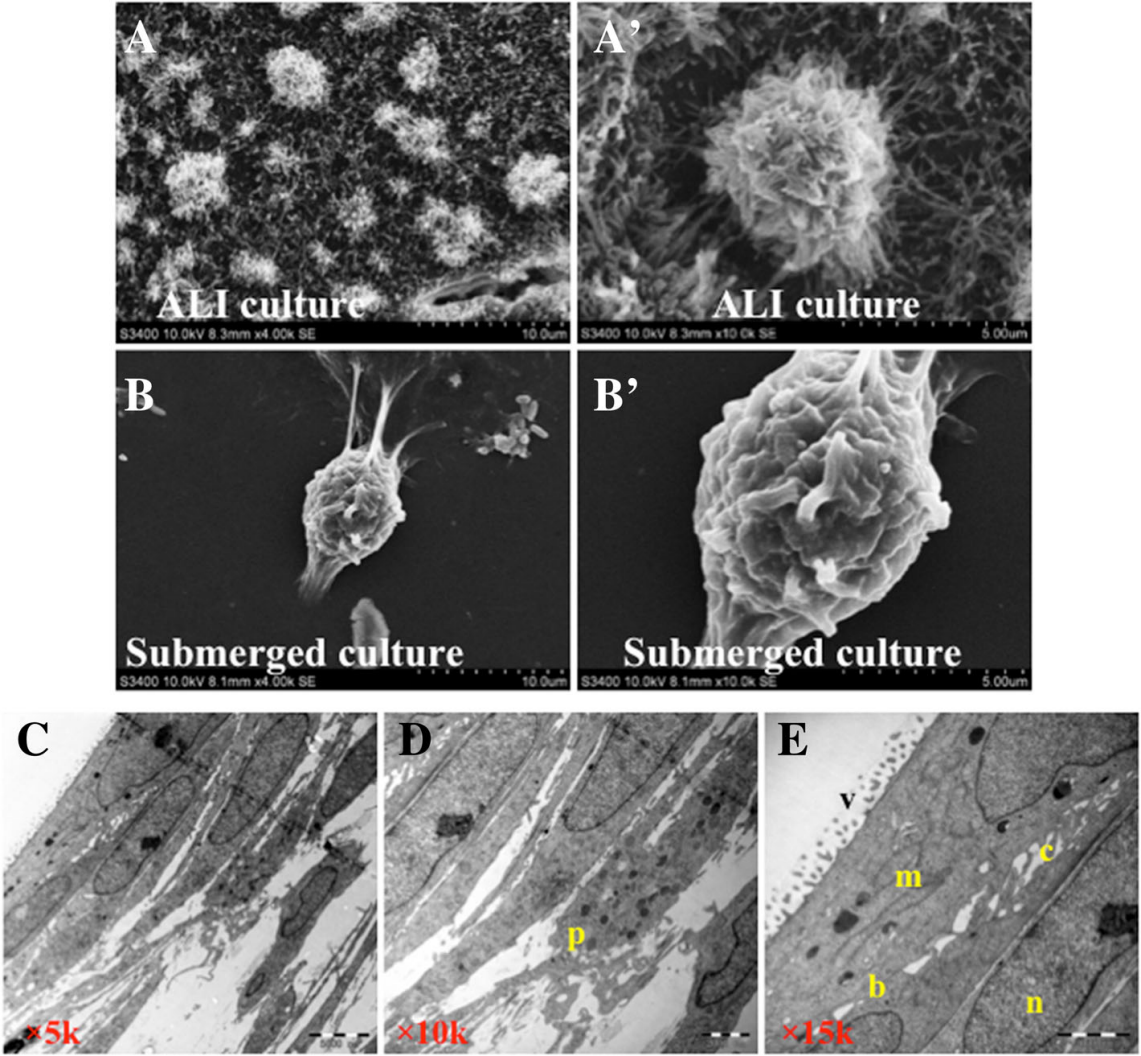
Morphological analysis of electronic microscopy. The passage 1 (P1) endometrial epithelial cells were cultured in an air–liquid interface (ALI) state for 2 weeks, and the ALI epithelial cultures and the P2 submerged monolayer cultures were employed for morphological analysis by scanning electronic microscopy (SEM) (**A**, **B**) and transmission electron microscopy (TEM) (**C**–**E**). (**A**, **B**) Representative images of SEM for endometrial epithelial cells cultured in an ALI state (A) and the logarithmic phase of submerged P2 cell culture (**B**). **A′** and **B′** were the higher magnifications of corresponding enlarged fields in **A** and **B**, respectively. Cells in submerged monolayer cultures showed a morphology of inerratic shapes with smooth surfaces, while ALI cultured cells exhibited anomalous shapes and rough cell surfaces with abundant secretions and microvilli on the surface of culture. (**C**–**E**) Representative TEM images of endometrial epithelial cells grown in ALI culture at magnification of 5,000× (**C**), 10,000× (**D**), and 15,000× (**E**) showed nucleus (n), microvilli (v), cilia (c), mitochondria (m), bridge (b), and secretory protein particles (p). Scale bars: 10 μm (**A** and **B**), 5 μm (**A′**, **B′**, and **C**), and 2 μm (**D** and **E**)

In order to characterize endometrial epithelial cells in an ALI state, P1 epithelial cells were seeded on collagen–pre-coated membranes of Millicell inserts and cultured in an ALI phase. Scanning electron microscopy showed anomalous shapes and rough cell surfaces with abundant secretion and microvilli on the surface of cells cultured in an ALI state (Fig. 2B), while monolayer cells cultured in the conventionally submerged condition displayed the morphology of inerratic shape with smooth surfaces (Fig. 2B). Transmission electron microscopy further unraveled that the structures of bridge, microvilli, and cilia and abundant secretory granules and mucus in ALI cultures (Fig. 2C–E) [8, 9].

### Immunological characterization of endometrial glandular epithelial cell ALI cultures

**Fig. 3:**
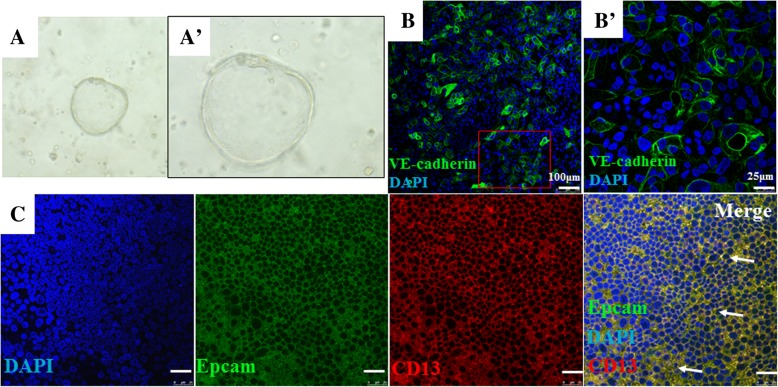
Whole mount immunofluorescent staining of endometrial epithelial cells cultured in an air–liquid interface (ALI) state and spheroid formation. The passage 1 (P1) endometrial epithelial cells were cultured in an ALI state for 2 weeks. (**A**) Human endometrial epithelial cells generated the spheroids by Matrigel sphere assay. (**A′**) A higher magnification of image (**A**). (**B**) Immunofluorescent staining for endometrial epithelial cell marker vascular endothelial-cadherin (VE-cadherin) (green). (**B′**) A higher magnification of image (**B**). (**C**) The co-expression of endometrial epithelial cell markers epithelial cellular adhesion molecule (EpCam) (green) and CD13 (red) was ascertained by immunofluorescence assay. Cell nuclei were counterstained with DAPI (blue). Arrows denote the co-localization of CD13 and EpCam staining (yellow). Scale bar = 25 μm

In order to further characterize endometrial epithelial cells in ALI cultures, the expression of cell-specific cell markers of endometrial epithelial cells was accessed in the whole mount membrane of Millicell by an immunofluorescent staining assay. This assay showed the epithelial cell marker vascular endothelial-cadherin (VE-cadherin) (Fig. 3B) and the co-localized expression of epithelial cell marker EpCam and stromal cell marker CD13 in cells cultured in an ALI state for 2 weeks (Fig. 3C). Together with aforementioned morphological data, this result suggested that endometrial epithelial cells held an epithelial stem/progenitor potential to differentiate into stromal-like cells in an ALI state. In order to investigate the capacity of cell differentiation, the P1 cells were resuspended in 50% BD Matrigel and cultured with modified medium, and the formation of spheroids was examined. As expected, the spheroid formation was observed in 14-day Matrigel cultures (Fig. 3A). This result further suggests that endometrial epithelial cells can develop spheroids with an epithelial stem/progenitor characteristic.

### Molecular characterization of endometrial glandular epithelial cell ALI cultures

**Fig. 4:**
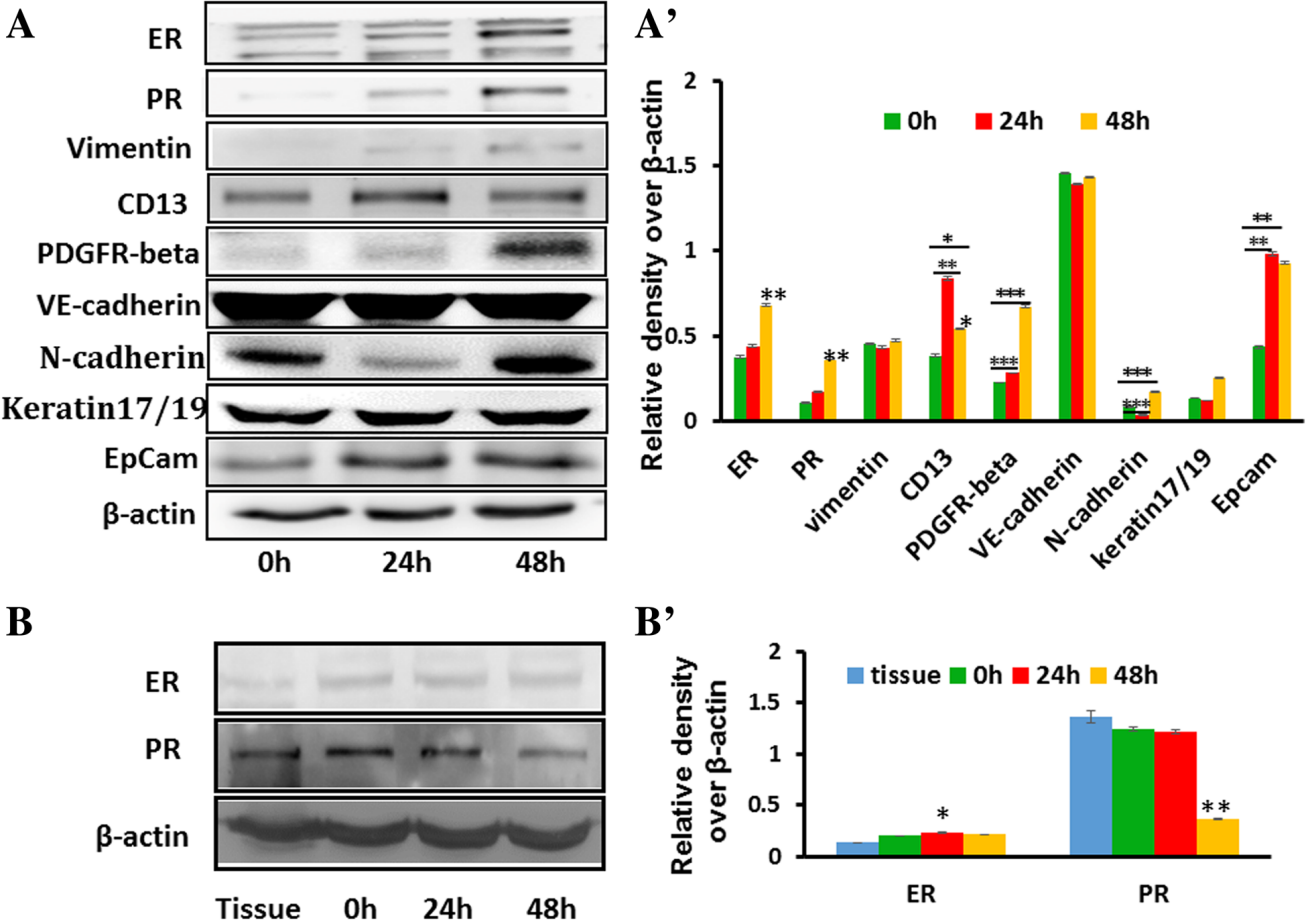
The expression of endometrial epithelial marker in air–liquid interface (ALI) cultured cells. The passage 1 (P1) endometrial epithelial cells cultured in an ALI state for 2 weeks (**A**) or in a submerged state (**B**) were treated with estrogen for indicated time points, and the cell lysates were analyzed by immunoblotting assay against indicated antibodies against proteins of interest. (**A**, **A′**) Representative blots showed a significantly induced expression of indicated endometrial epithelial cell markers of ALI culture cells in response to estrogen (**A**). (**A′**) Semi-quantitative analysis of fold changes of the expression of indicated proteins in (**A**) accessed by a densitometric assay. (**B**, **B′**) Representative blots showed the expression of estrogen receptor (ER) and progesterone receptor (PR) of submerged cell cultures in response to estrogen (**B**). (**B′**) Semi-quantitative analysis of fold changes of the expression of proteins of interest in (**B**) accessed by a densitometric assay. Compared with cells cultured in the absence of estrogen, *P <0.05; **P <0.01; ***P <0.0001 (analysis of variance)

In normal endometrium, estrogen stimulates the proliferation of endometrial glandular epithelial cells in the basal endometrium, we thus further investigated the characterization of the effect of estrogen on the expression of estrogen receptors (ERs), epithelial and stromal markers in cells cultured in the ALI state. Interestingly, an exposure of progesterone exhibited an increased expression of ER, progesterone receptor (PR), epithelial cell marker N-cadherin, EpCam, and stromal cell markers CD13, PDGFR-beta, and Vimentin in ALI cultured cells (Fig. 4A). However, epithelial cell marker Keratin 19 and VE-cadherin were not/19 and VE-cadherin were not altered in cells cultured in the ALI state. However, the addition of progesterone did not affect or inhibit the expression of ER and PR in endometrial epithelial cells cultured in a monolayer submerged state (Fig. 4B). This result implies that the characteristics of differentiated ALI human endometrial cell culture may be a reliable and feasible model able to mimic human endometrium *in vitro*.

## Discussion and conclusion

In this report, we described the isolation and expansion culture of human endometrial epithelial cells and the characteristics of endometrial epithelial cells cultured in a three-dimensional (3D) ALI state. We showed that a subset of endometrial epithelial cells had potential for spheroid formation, epithelium regeneration, and differentiation into stromal-like cells. This study thus introduces a useful approach for efficient isolation and expansion of human endometrial epithelial cells in stem cell biology research and possibly in studies of autologous cell transplantation therapy for endometrial injury diseases. In addition, the human endometrial epithelial ALI culture may be a feasible and reliable model for investigating the biological characteristics and mechanisms of endometrial epithelial cells or endometrial diseases *in vitro*. Together with the capacity of epithelial reconstitution and stromal cell differentiation in the ALI state demonstrated by others, our results imply that a subset of endometrial epithelial cells may retain their stem/progenitor cell potency for proliferation and differentiation.

The endometrium is a highly and cyclically regenerating organ by regulating hormones [10]. Estrogen plays an important part in the development and regeneration or repair of injured endometrium [11, 12]. In this context, estrogen could stimulate the proliferation of endometrial epithelial cells at every menstrual cycle in a normal endometrium by binding to ER and PR [13]. In this study, we also demonstrated an induced augmentation of ER and PR along with the increased expression of EpCam, N-cadherin, and CD13 in human ALI endometrial epithelial culture in the presence of progesterone. These data imply that progesterone-promoted proliferation and differentiation of endometrial epithelial cells occur in ALI endometrium, which is similar to the response of endometrial epithelial cells *in utero in vivo*, suggesting the reliability and feasibility of ALI endometrial epithelium as an *in vitro* 3D model for mimicking endometrial epithelium *in vivo*.

In conclusion, this report described methods for the isolation and expansion of human endometrial epithelial cells and generation of human endometrial ALI epithelium. The ALI culture may offer a reliable and feasible model for biomedical research and stem cell biology studies of human endometrium *in vitro*. However, the limited passages of primary endometrial epithelial cells using ROCK inhibitor–modified medium is a methodological limitation of this study, and further optimizations of culture media or conditions for unlimited culture are required for future autologous endometrial epithelial cell transplantation research *in vivo*.

## Additional files

Additional file 1: Detail materials and methods. (PDF 359 kb)

Additional file 2: Supplementary table of antibodies used for immunostaining in this report. (PDF 128 kb)

## Abbreviations

3D: Three-dimensional
ALI: Air–liquid interface
EpCam: Epithelial cellular adhesion molecule
ER: Estrogen receptor
OCT 3/4: Octamer-binding transcription factor 3/4
P: Passage
PBS: Phosphate-buffered saline
PR: Progesterone receptor
ROCK: Rho-associated protein kinase
SOX2: SRY-Box 2
SSEA-1: Stage-specific embryonic antigen-1
