# Supplementary material for: Development and characterization of a polarized human endometrial cell epithelia in an air–liquid interface state
Source: Stem Cell Res Ther. 2018 Aug 9;9:209. doi: 10.1186/s13287-018-0962-6 (PMC6085666; doi:10.1186/s13287-018-0962-6)
Supplement: Supplementary file 1 — Detail materials and methods. (PDF 359 kb) [file 13287_2018_962_MOESM1_ESM.pdf]

Additional file 1

MATERIAL AND METHODS

Tissue collection

Biopsies of human endometrium samples were obtained from the premenopausal women undergoing hysteroscopy in General Hospital of Ningxia medical university. Tissues from twelve donors were analyzed included in this study. The endometrium was scraped off and collected into D-Hanks Phosphate Buffer Solution at 4°C, and subsequently isolated cell within 2-4 h. The sample was collected with informed patient consent and Ethics Committee approval.

Isolation and culture of human endometrial glandular epithelial cells

The isolation of human endometrial epithelial cells was conducted as described in a previous study with minimal modification [1]. Briefly, the human endometrial biopsy was minced with scissors into small pieces of less than 1 mm^3, before it was washed with D-Hanks Phosphate Buffer Solution (PBS) containing 100 U/ml penicillin, 100 mg/ml streptomycin, 2.5 mg/ml amphotericin B. Subsequently, the minced endometrial biopsies were dissociated in dissociation buffer containing 3 mg/ml collagenase type 4 (Sigma, USA) in PBS for 7 min at 37˚C with gentle agitation. Then, the same volume of Accumax (Gibco, USA) was added in the dissociated solution and incubated for additional 7 minutes at 37˚C with continuous agitation. The digestive reaction was terminated by adding fetal bovine serum (FBS) to the dissociated fragments/cell suspension in a final concentration of 10%. The dissociated fragments/cells suspension was then filtered through a 400 mesh nylon sieve, the residual cell clumps on the sieve were glandular epithelial cells and were washed off with D-Hank's into a tube. The cells were collected by centrifugation (100 × g for 5 min), and resuspended in 2 ml of DMEM, 7% FBS, 10 μM of Rho-associated protein kinase (ROCK)-inhibitor Y-27632 (Sigma, St. Louis, MO, USA), 100 U/ml penicillin, 100 mg/ml streptomycin, 2.5 mg/ml amphotericin B and seeded onto a 10-cm culture dish pre-coated with 70 μg/ml collagen type I rat tail (BD

Biosciences, USA). The cells was maintained in the culture medium at 37°C in a humidified environment with 5% CO_2. The adherent cells were dissociated using Accutase solution (Millipore) at 2-3 days after seeding, and the cells were reseeded at a ratio of 1:3-5 for passage.

Generation of air-liquid interface (ALI) model of endometrial epithelial cells

Above Passage 1 (P1) primary endometrial epithelial cells were employed for the generation of the ALI epithelial cell model in vitro as described elsewhere [2]. For generation of ALI cultures, single cell suspension were seeded on the apical surfaces of Millicell inserts (0.4mm pore, polycarbonate, Millipore, Billerica, MA, USA) that were pre-coated with collagen type I at densities of 1.8×10^6 and 0.32×10^6 cells per well for diameters of 30 mm and 12 mm inserts, respectively. The culture medium at apical side was removed at 24 h after the seeding and the medium of bottom was exchanged with 2% Ultroser G (USG) medium (Pall, USA) to establish an ALI condition. The ALI cultured cells were refreshed with medium in the bottom chamber of inserts with 2 days intervals. For estrogen stimulation, cell cultures after 2 weeks were exposed in medium containing 1 μM of progesterone (Sigma, St. Louis, MO, USA) for indicated time periods.

Spheroid culture for human endometrial epithelial cells

Above passage 1 (P1) primary endometrial epithelial cells were used for the generation of spheroid cell model *in vitro*. The P1 cells were resuspended in 50% BD Matrigel /10%DMEM/12 containing 1 μM insulin, 1 μM A-83-01(TGF antagonist), 1 μM DMH-1, 1 μM CHiR99021 and 25 ng/mL EGF. After culture 3-4 d, the culture medium was exchanged with Ultroser G (USG) medium (Pall, USA) and continued culture 10 days.

Immunocytochemistry staining and immunofluorescent staining

For Immunocytochemistry (ICC) and immunofluorescent (IF) staining, cells cultured on cover slides or membranes of Millicell inserts were fixed by 4% paraformaldehyde at room temperature (RT) for 20 min and rinsed three times with 1 × PBS for 5 min each, before they were permeabilized with 0.2% Triton X-100 for 15 minutes, and incubated with 5% normal horse serum in Blocking Buffer (Thermos

scientific, Germany) in PBS for 30 min in a humidified chamber. The cells were then incubated with primary antibodies to epithelial cell marker EpCam (1:200, 63316-1-Ig, Proteintech), VE-cadherin (1:200, 36-1900; ThermoFisher) or stroma cell marker CD13 (1:200, 14553-1-AP, Proteintech), Ki67 (1:200, 14-5698-82, Invitrogen) in PBS at 4 °C overnight, and subsequently incubated with an appropriate secondary antibody conjugated with horseradish peroxidase (HRP) for ICC staining, or fluorescence dyes for IF staining (All secondary antibodies were products of Jackson Immunoresearch and were diluted at 1:500 for use) for 2 h. For ICC, the signal was visualized after the slides were incubated with the DAB substrate. Images were acquired by Leica TCS SP2 A0BS Confocal System and processed them on Leica Confocal Software v.2.6.1 (Leica, Germany). Hematoxylin was used for counterstaining in ICC, and DAPI was used for nuclear labeling for IF.

Electron Microscopy

Scan electron microscopy (SEM) and transmission electronic microscopy (TEM) were applied to evaluate the morphology of the membranes of two week-ALI cultures. For SEM, the endometrial cells of two week-ALI cultures were rinsed with PBS and fixed in 3.5% glutaraldehyde in 0.15 M phosphate buffer, pH 7.4. After 1 h, the membrane was dehydrated in a graded series of hexamethyldisilazane and stained with platinum/palladium. Ultrathin sections were cut with glass knives and collected images on a Hitachi S-3400 Microscope (Hitachi LTD, Japan). For TEM, the cells were fixed same as SEM, followed by infiltration with Spurr resin following dehydration. Serial sections of 80 nm were then viewed on a Hitachi H-7000 Electron Microscope (Hitachi LTD).

Immunoblotting Analysis

The cells were rinsed in PBS and lysed by homogenizing cells in RIPA buffer (50mMTris-HCl, pH 7.5, 5mMEDTA, 150mMNaCl,and 0.5% NP-40) for 3.5 h on ice. Then the cell lysate was collected by centrifuge tube, centrifuged at 12,000 ×g for 30 min at 4 °C. The resultant supernatants were harvested as whole cell extracts. Approximately 40 μg protein was resolved in a 10% sodium dodecyl sulfate- (SDS-) polyacrylamide gel (SDS-PAGE) and transferred to a PVDF membrane (Millipore,

Billerica, MA, USA). The membrane was blocked with 5% fat-free dry milk in PBS containing 0.2% Tween-20 for 1 h at room temperature. Subsequently, incubated with primary antibodies at 4°C overnight. The membranes were incubated with peroxidase-conjugated secondary antibody prior to be detected with ECL plus western blotting detection reagents. The levels of protein expression were semi-quantitative by optical densitometry using ImageJ Sotware version 1.46 (https://rsb.info.nih.gov/ij/). The ratio of the net intensity of each sample divided by the β-actin internal control was calculated as densitometric arbitrary units (AU), a fold of change AU was served as an index of relative expression of a protein of interest. The primary antibodies used immunoblotting assay were rabbit anti-ER (estrogen receptor) (1:1000, 21244-1-AP, Proteintech), PR (progesterone receptor) (1:1000, Merck Millipore, USA), Cytokeratin 17/19 (1:1000, 10143-1-AP, Proteintech), CD13 (1:1000, 14553-1-AP, Proteintech), mouse anti- EpCam (1:1000, 63316-1-Ig, Proteintech).

Statistical analysis

The data were obtained from at least three independent experiments for each experimental condition and presented as mean ± standard error of the mean (SEM), and their statistical significance was analyzed by one-way ANOVA.

References

1. Masuda A, Katoh N, Nakabayashi K, Kato K, Sonoda K, Kitade M, Takeda S, Hata K, Tomikawa J. An improved method for isolation of epithelial and stromal cells from the human endometrium. J Reprod Dev. 2016, 62(2):213-218.
2. Liu X, Driskell RR, Engelhardt JF. Stem cells in the lung. Methods Enzymol. 2006, 419:285-321.
