# Supplementary material for: Development and characterization of a polarized human endometrial cell epithelia in an air–liquid interface state
Source: Stem Cell Res Ther. 2018 Aug 9;9:209. doi: 10.1186/s13287-018-0962-6 (PMC6085666; doi:10.1186/s13287-018-0962-6)
Supplement: Supplementary file 2 — Supplementary table of antibodies used for immunostaining in this report. (PDF 128 kb) [file 13287_2018_962_MOESM2_ESM.pdf]

Table S1. Antibodies used for immunostaining in this report

Protein target	Full name of antibody	Manufacturer, catalog #	Species raised in; monoclonal or polyclonal	Dilution
SSEA-1	Stage-specific Embryonic Antigen-1	R&D Systems, MAB2155	Mouse; Monoclonal	1 to 200
Nanog		Abcam, ab80892	Rabbit; Polyclonal	1 to 200
PCNA	Proliferating Cell Nuclear Antigen	Millipore, MABE288	Mouse; Monoclonal	1 to 200
p63	Transformation-related Protein 63	Thermo Scientific, MS-1084	Mouse; Monoclonal	1 to 200
OCT-3/4	octamer-binding transcription factor 3/4	R&D Systems, MAB1759	Monoclonal; Rat	1 to 200
Ki67	Antigen KI-67	Thermo Scientific, RB-9043	Rabbit; Polyclonal	1 to 200
SOX2	SRY (sex determining region Y)-box 2	R&D Systems, MAB2018	Mouse; Monoclonal	1 to 200
CD117	proto-oncogene c-Kit	DAKO, A4502	Rabbit; Polyclonal	1 to 200
CD133	prominin-1,	Abcam, ab19898	Rabbit; Polyclonal	1 to 200

Secondary Antibody	Fluorochrome	Manufacturer, catalog #, and/or name of individual providing the antibody	Species raised in; monoclonal or polyclonal	Dilution used
Anti- rabbit IgG (H+L)	Alexa Fluor® 448,	Jackson Immunoresearch; 109010	Donkey; Polycolonal	1 to 500
Anti- mouse IgG (H+L)	Alexa Fluor® 448	Jackson Immunoresearch; 108266	Donkey; Polycolonal	1 to 500
Anti- Rat IgG (H+L)	Alexa Fluor® 448,	Jackson Immunoresearch; 109912	Donkey; Polycolonal	1 to 500
